# A high-sensitivity MEMS gravimeter with a large dynamic range

**DOI:** 10.1038/s41378-019-0089-7

**Published:** 2019-10-07

**Authors:** Shihao Tang, Huafeng Liu, Shitao Yan, Xiaochao Xu, Wenjie Wu, Ji Fan, Jinquan Liu, Chenyuan Hu, Liangcheng Tu

**Affiliations:** 1 MOE Key Laboratory of Fundamental Physical Quantities Measurement & Hubei Key Laboratory of Gravitation and Quantum Physics, PGMF and School of Physics, Huazhong University of Science and Technology, 430074 Wuhan, PR China; 2 Institute of Geophysics, Huazhong University of Science and Technology, 430074 Wuhan, PR China

**Keywords:** Electrical and electronic engineering, Optical physics

## Abstract

Precise measurement of variations in the local gravitational acceleration is valuable for natural hazard forecasting, prospecting, and geophysical studies. Common issues of the present gravimetry technologies include their high cost, high mass, and large volume, which can potentially be solved by micro-electromechanical-system (MEMS) technology. However, the reported MEMS gravimeter does not have a high sensitivity and a large dynamic range comparable with those of the present commercial gravimeters, lowering its practicability and ruling out worldwide deployment. In this paper, we introduce a more practical MEMS gravimeter that has a higher sensitivity of 8 μGal/√Hz and a larger dynamic range of 8000 mGal by using an advanced suspension design and a customized optical displacement transducer. The proposed MEMS gravimeter has performed the co-site earth tides measurement with a commercial superconducting gravimeter GWR iGrav with the results showing a correlation coefficient of 0.91.

## Sensors: A MEMS gravimeter combines high precision with portability

Researchers in China have developed a small, portable gravimeter based on micro-electromechanical-system (MEMS) technology with a sensitivity and dynamic range comparable with larger, commercially available gravimeters. The new device was created by a team led by Liangcheng Tu at Huazhong University of Science and Technology. The MEMS mechanism consists of a spring-mass system designed around a combination of curved and folded beams. Together, these ensure that the system is stiff under low loads but flexible at loads around 1 g. An optical component measures the displacement of the proof mass to measure gravitational acceleration. The team tested their design alongside a commercial gravimeter and found a 90% correlation in the measurements of Earth tides, demonstrating its utility for applications from oil and gas exploration to hazard detection.

## Introduction

Since the famous free-fall experiments were demonstrated at the Leaning Tower of Pisa in the 1600s, the conventional standard value of the gravitational acceleration near the Earth’s surface, denoted as 1 *g*, has been gradually accepted by the public as 9.8 m/s^2^ (980 Gal, 1 Gal = 1 cm/s^2^)^1^. However, in fact, the gravitational acceleration, which represents the intensity of the gravitational field, varies with both location and time since it is determined by both the gravitational forces of the Earth and other celestial bodies and the centrifugal force due to Earth’s rotation^2^. For a certain area of interest, the location-related portion of the gravitational acceleration is determined by the local mass distribution, latitude, and longitude, while the time-related portion is determined by tides, crustal movement, and other time-dependent phenomena^3^. Hence, precise measurement of the gravitational acceleration is of great significance in many fields such as geophysics^4,5^, oil, gas and mineral exploration^6^, water-floor monitoring^7,8^, and hazard forecasting^9,10^.

There are several commercial systems that are effective for these applications, and they can be divided into two categories: absolute gravimeters and relative gravimeters. Absolute gravimeters determine the absolute values of the gravitational acceleration by directly measuring the acceleration of a freely falling body. The freely falling body can be either a microscopic object, such as the group of atoms used in atom interferometry-based gravimeters^11–14^, or a macroscopic object, such as the prisms used in the Micro-g FG-5^15^. Relative gravimeters determine the relative variations in the gravitational acceleration by measuring the displacement of the proof mass, which is suspended by a spring, such as in the Scintrex CG-5^16^, or by superconductive levitation, such as in the GWR superconducting gravimeter^17^. Recently, a quartz resonant accelerometer has been used to measure gravitational acceleration^18^. Absolute gravimeters are usually used in monitoring stations or as a reference gravimeter to calibrate relative gravimeters. Compared with absolute gravimeters, relative gravimeters are much smaller and lighter and generally have a better reading resolution. Therefore, they are commonly used for mapping regional gravitational fields^19^. However, relative gravimeters have to be very stable and the inherent drifts have to be calibrated by measuring time-dependent gravity changes such as earth tides. Apart from the stability, the other main challenge of developing a relative gravimeter lies in the difficulty of detecting very small gravity changes (a few µGal) under the much larger bias gravity (980 Gal) on Earth. As the most commonly used relative gravimeter, the CG-5 gravimeter can achieve a reading resolution of 0.1 μGal within 5 min and a standard deviation of less than 5 μGal. However, it costs over one hundred thousand dollars and weighs 8 kg; therefore, it is not fit for large-scale deployment.

Recently, micro-electromechanical-system (MEMS) technology, which was originally used to batch produce low-cost sensors and actuators, has been shown to be capable of developing very sensitive accelerometers^20^. For example, a few capacitive accelerometers with a sensitivity of less than 20 μGal/√Hz have been reported^21–23^, an optical accelerometer with a sensitivity of 17 μGal/√Hz has been developed^24^, a MEMS electrochemical seismic sensor with a noise level of 3.2 μGal/√Hz has been developed^25^, and some high-precision resonant MEMS accelerometers have been reported^26–30^. A MEMS seismometer with a sensitivity of 0.3 μGal/√Hz has been developed by researchers from Imperial College and landed on Mars by the NASA’s InSight mission^31,32^. Researchers from the University of Glasgow demonstrated a MEMS gravimeter with an ultra-low resonant frequency of 2.3 Hz and a sensitivity of 40 μGal/√Hz using a geometrical anti-spring suspension and an optical shadow displacement transducer^33,34^. However, this MEMS gravimeter is 20 times less sensitive and has a smaller dynamic range than the commonly used CG-5 relative gravimeter, which restricts its application in the field. Hence, the issues of low sensitivity and small dynamic range need to be addressed.

In this paper, we introduce a more practical MEMS gravimeter that has a higher sensitivity and a larger dynamic range and is promising for worldwide deployment. In addition, we conduct co-site earth tide measurements by using both the proposed MEMS gravimeter and a superconducting gravimeter.

## Results

### Model of the MEMS mechanism

**Fig. 1:**
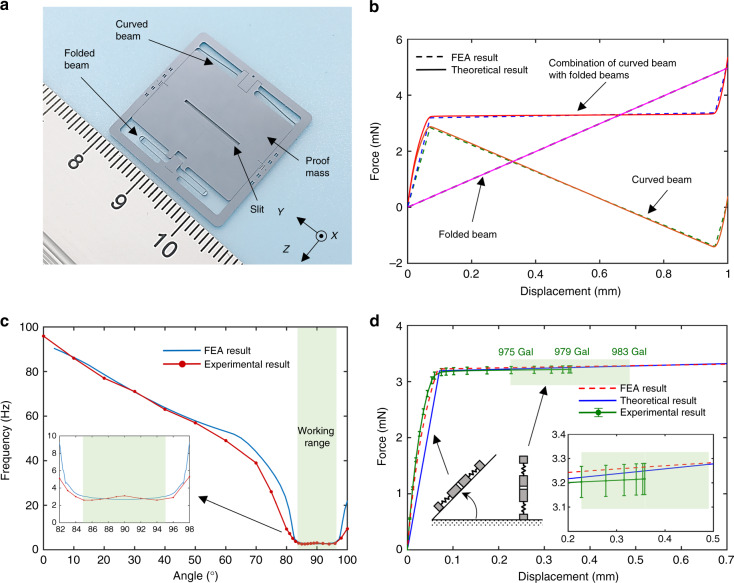
The MEMS mechanism design. **a** The MEMS mechanism is a spring-mass system with a proof mass suspended by a curved beam and two folded beams. A slit is located in the centre of the proof mass. **b** The force-displacement curves of different springs are plotted. **c** The resonant frequency decreasing behaviour with tilt is plotted. The resonant frequency will significantly decrease from 95 Hz to 2.6 Hz (85°) when the curved beam exhibits a negative stiffness. The resonant frequency will remain at ~3.1 Hz when the mechanism is vertical. **d** The experimental force-displacement result is plotted along with the FEA result and the theoretical result for comparison (according to ref. ^35^, the gravitational acceleration of our laboratory is 979 Gal)

Figure 1a illustrates the fabricated MEMS mechanism, which is a silicon-based spring-mass system with dimensions of 25 × 25 × 0.4 mm^3^. The proof mass is suspended by both a curved beam and folded beams on each side, allowing the mass to move laterally in-plane. These beams are designed with a width of 30 μm and a thickness of 400 μm considering the processing capability. In the middle of the proof mass, there is a slit with cross-sectional dimensions of 12 × 0.5 mm^2^ to construct the optical displacement transducer.

The essence of achieving an ultra-sensitive gravity measurement mechanism with a large dynamic range that can work under 1 *g* and work worldwide lies in a careful suspension design. The design rational is that the suspension should be stiff when the loading gravity component is less than 1 *g* and become compliant when the loading is close to 1 *g*. In addition, it must have a reasonably large working range for measuring the location-dependent variations all over the world. Therefore, the details of the suspension design are described below. The curved beam has a cosine shape, exhibits a highly nonlinear force-displacement behaviour and has been applied in switches^36^. The basis of the nonlinear behaviour is the bi-stable mechanism. With the clamp-clamp condition, when the centre of the beam moves laterally, the elastic force of the beam is not always proportional to the displacement. Generally, as the displacement increases, the elastic force increases. When the displacement reaches a specific point, the elastic force will decrease as the displacement increases. After another specific point, the elastic force will again increase as the displacement increases. Therefore, the stiffness of the beam can be approximately described as three segments: two positive stiffness segments near the two ends and one negative stiffness segment in the middle. The energy stored inside the deflected curved beam includes both the bending energy and the axial compression energy. The bending energy increases monotonically as the displacement increases, while the compression energy reaches the maximum value at the turning points and then decreases after crossing these points. The curved beam was designed to have a faster decrease in the compression energy than increase in the bending energy after crossing the turning points, resulting in a negative force. Therefore, the stiffness of the curved beam behaves as positive-negative-positive in the three segments. However, the negative stiffness introduces the snap-through instability and even results in damage to the compliant mechanisms. Therefore, the folded beams, which have a positive stiffness and obey Hooke’s law, are introduced into the suspension to compensate for the negative stiffness. With the careful geometric design, the developed suspension only has positive stiffness and provides a quasi-zero positive stiffness over a wide displacement range. Theoretical values for the curved beam, the folded beams and the entire suspension were determined and are plotted in Fig. 1b. In addition, nonlinear finite element analysis (FEA) was conducted, which agrees well with the theoretical results. The results indicate that the suspension stiffness decreases to quasi-zero as the loading force increases and remains at this value over a large displacement range, which is promising for meeting the demands of both a high sensitivity and a large dynamic range.

A static force loading experiment was conducted by tilting the MEMS mechanism. As the tilt angle increases, the gravity component applied on the MEMS mechanism increases up to 1 *g*. The measured fundamental frequencies of the MEMS mechanism vary from 95 Hz in the horizontal arrangement to 3.1 Hz in the vertical arrangement, and the minimum frequency is 2.6 Hz at 85 degrees, as shown in Fig. 1c. Within the tilt range between 85 degrees and 95 degrees, the fundamental frequencies have a mean value of 2.9 Hz and a standard deviation of 0.2 Hz, and this range is defined as the working range of the designed MEMS mechanism. Figure 1d illustrates the FEA and experimental results, showing that in the working range, the measurable gravitational acceleration values range from 975 Gal to 983 Gal. According to ref. ^37^, the local gravitational acceleration at different locations on Earth varies from 976 Gal to 982 Gal, which suggests that the designed MEMS mechanism is promising for worldwide application.

In addition, a ring-down test was performed in which the MEMS mechanism was tilted to be vertical. Based on the experimental results, a quality factor of ~1200 at a pressure of 1 Pa is obtained. The thermal equivalent acceleration noise (NEA) floor can be given by

1 $${\mathrm{NEA}} = \sqrt {4k_bT\frac{{\omega _0}}{{mQ}}} ,$$

where *k*_*b*_ is the Boltzmann constant, *T* is the temperature in Kelvin, *ω*_0_ is the resonant frequency, *m* is the proof mass, and *Q* is the quality factor. In this case, at room temperature (25 °C), the thermal equivalent acceleration noise floor of the MEMS mechanism is 0.08 μGal/√Hz. Therefore, this MEMS mechanism could be very sensitive to acceleration variations under 1 *g*.

### Optical displacement transducer

**Fig. 2:**
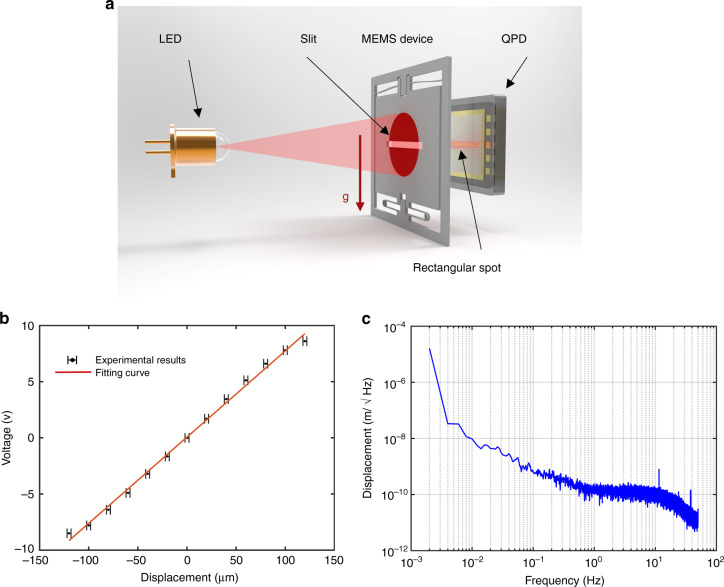
The MEMS device. **a** The working principle of the MEMS device. **b** Calibration of the optical displacement sensor. **c** The noise performance of the optical displacement sensor

A customized high-sensitivity optical displacement transducer was employed to measure the proof mass displacement due to acceleration. The main components of the optical displacement transducer are a light-emitting diode (LED), a quadrant photodiode (QPD) and the slit of the proof mass in between, as shown in Fig. 2a. Collimated light emitted from the LED is shot onto the QPD through the 0.5 mm wide slit. A conditioning circuit of the optical displacement transducer was designed with a zero voltage output at the nominal position where all three main components are centrally aligned. When the proof mass moves, the output of the transducer will linearly vary within a certain displacement range.

A series of experiments were performed to evaluate the optical displacement transducer. First, a piece of silicon with the same profile as the proof mass was fixed on a motorized positioning stage in front of the QPD. The silicon piece was driven over a large travel range with a step of 20 µm. The corresponding output of the displacement transducer was recorded and then plotted, as shown in Fig. 2b, with the results showing a nonlinearity of less than 7% in a working range of 240 μm. Then, the silicon piece was positioned at the nominal point to evaluate the noise floor of the optical displacement transducer. The experimental results shown in Fig. 2c suggest that the noise floor of the displacement transducer is 0.2 nm/√Hz at 1 Hz. In this case, for a MEMS mechanism with a fundamental frequency of 3.1 Hz under 1 *g*, the electrical equivalent acceleration noise floor of the MEMS device can be calculated as 7 μGal/√Hz at 1 Hz, and the dynamic range can be 8000 mGal.

### Calibration and characterization

**Fig. 3:**
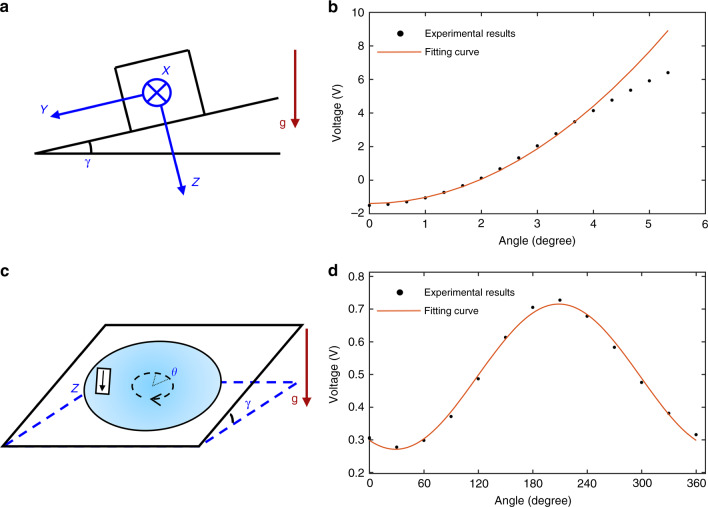
Calibration experiment. **a** The calibration experiment. **b** The calibration results of the MEMS device. **c** The 12-point rotation experiment. **d** The calibration results of the cross-axis sensitivity

The MEMS device was calibrated by the tilt method, as shown in Fig. 3a. A biaxial dividing head with a precision of 10 arcseconds was applied to change the component of gravity along the device’s sensitive axis. In this experiment, the MEMS device was fixed on the dividing head with the sensitive axis (*Z*-axis) perpendicular to the mounting surface. After the mounting surface was levelled, the device was tilted by 20 arcminutes about the *X*-axis each time. The calibration results are shown in Fig. 3b. By analysing the data with the fitting formula

2 $${\mathrm{Output}} = b + K_z \cdot \left( {1 - \cos (\gamma )} \right)$$

where *b* is the bias and γ is the angle between the mounting surface and the horizontal plane, the scale factor (*K*_*z*_) of the MEMS device is determined to be 2.2 mV/mGal. The nonlinearity of the output mainly arises from the nonlinear behaviour of the suspension and the nonlinearity of the optical displacement transducer. This nonlinearity could be calibrated and compensated by a look-up table.

Then, the cross-axis sensitivity of the MEMS device was calibrated by a 12-point rotation experiment, as shown in Fig. 3c. The output varied with the cross-axis input acceleration, which was modulated by the rotation angle. After tilting to a certain angle, such as 2°15′, the device was rotated about the *Z*-axis to 12 points. Due to some imperfections of the MEMS mechanism and misalignment, the output will change with the rotation angle, as shown in Fig. 3d. The output formula can be given by

3 $${\mathrm{Output}} = b + K_x \cdot \sin (\gamma ) \cdot \sin (\theta ) + K_y \cdot \sin (\gamma ) \cdot \cos (\theta )$$

where *θ* is the rotation angle*. K*_*x*_ and *K*_*y*_ can be obtained as 2.7 μV/mGal and 4.9 μV/mGal. The *X*-axis and *Y*-axis sensitivity are 1.3 × 10^−3^ μGal/μGal and 2.2 × 10^−3^ μGal/μGal, respectively. (1 × 10^−3^ μGal/μGal means that a cross-axis acceleration change of 1 μGal will lead to a change of 1 × 10^−3^ μGal in the output).

**Fig. 4:**
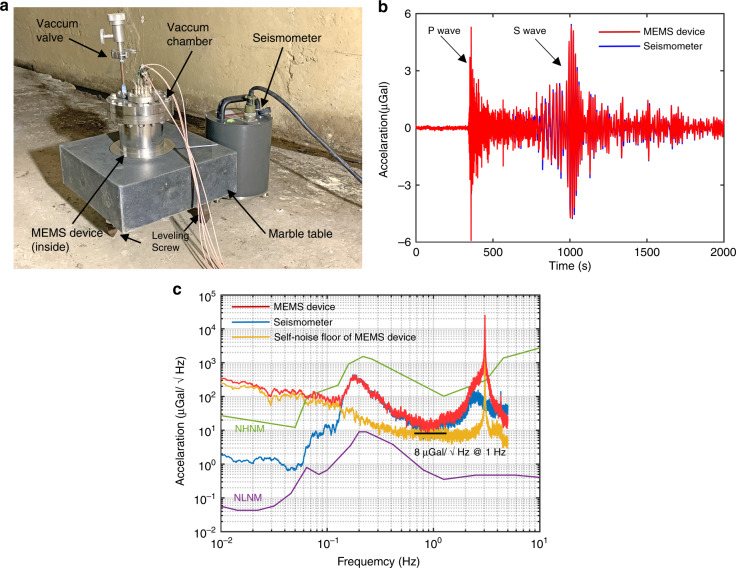
Calibration and noise performance. **a** The setup of the MEMS device in the cave laboratory. **b** The tele-seismic signal caused by the earthquake that occurred in Hokkaido, Japan. The data were sampled at 1 Hz. **c** The acceleration power spectral density of the MEMS device and the seismometer (both sampled at 10 Hz) are plotted to present the noise performance. The red line represents the MEMS device, the blue line represents the seismometer, and the yellow line represents the self-noise floor of the MEMS device with the seismic noise eliminated. The minimum noise floor is ~8 μGal/√Hz at 1 Hz, while the resonant frequency is 3.1 Hz

The evaluation of the self-noise floor of high-precision accelerometers generally requires a quiet place. However, the predicted self-noise floor of the developed MEMS device is lower than the ground noise floor according to Earth’s new high noise model (NHNM)^38^; therefore, the self-noise cannot be directly characterized under the large background seismic noise. Hence, coherence analysis^39^ was introduced to solve this issue. The experimental setup is shown in Fig. 4a. Both the MEMS device, which was sealed in a vacuum chamber, and a reference commercial seismometer, CMG-3ESPC, were placed in a cave laboratory where the temperature change was within 100 mK per day. Both instruments detected the same signals since the sensitive axes of both instruments were aligned. During the long-term data recording, a few sets of unusual data were recorded. According to the data of the reference seismometer, some of the recordings were tele-seismic signals. Figure 4b shows the seismic signal caused by the earthquake occurring in Hokkaido, Japan on 5 September 2018. The arrivals of the P and S waves can be clearly distinguished, and the correlation coefficient of the data measured by these two instruments is 0.98. Unlike seismic signals, earth tremors are periodic signals with lower frequencies; therefore, earth tremors are generally observed in the frequency domain. The acceleration power spectral density of both the MEMS device and the seismometer are shown in Fig. 4c. The earth tremor peaks are evident at ~0.2 Hz. Based on the correlation measurements, the output of the MEMS device divided by the output of the commercial seismometer can be used to obtain the scale factor of the MEMS device. The calibration results show that the scale factor of the MEMS device is the same as the result calibrated by the tilt method. With the earth tremor signal eliminated by correlation analysis, the measured self-noise floor of the MEMS device is 8 μGal/√Hz at 1 Hz.

There are several possible error sources in the MEMS gravimeter readings, listed in Table 1^40^.

**Table 1:** Summary of the main error sources of the MEMS gravimeter

Error sources	Characteristic	Correction
Linear creep of structure	Elastic relaxation. (DC~)	Linear trend removal from the data.
Noise from electronics	Noise from LED, QPD and other electronics. (DC~)	Averaging and filtering.
Temperature change	Temperature-dependent properties, such as Young’s modulus and sensitivity of optical devices. (DC~)	Temperature control and temperature effect correction.
Seismic noise	Man-made and natural sources. (>0.1 Hz)	Averaging and filtering.
Wind-induced vibration	Unexpected airflow. (>0.5 Hz)	Windshield, averaging, and filtering.
Atmospheric pressure change	Change of air buoyancy.	Vacuum sealed.

### Earth tide measurement

**Fig. 5:**
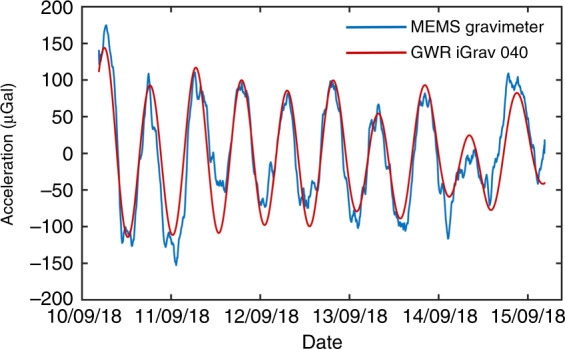
The Earth tides. The comparison measurements of the Earth tides acquired by the MEMS device and the superconducting gravimeter. The red line was obtained by the superconducting gravimeter, and the blue line was obtained by the MEMS device. The data were averaged with a time constant of 60 min. The correlation coefficient of these two sets of data is 0.91. (The original data are shown in Fig. 6a)

The MEMS device has been located on the ground of the cave laboratory for continuous recording without vibration isolation or vacuum maintenance since September 2018. A commercial superconducting gravimeter, GWR iGrav, was used as a reference. The blue line in Fig. 5 shows the data recorded by the MEMS device for 5 days from 10 to 15 September 2018 after data processing. Based on a comparison with the data (in red) acquired by the GWR iGrav superconducting gravimeter, the strong correlation coefficient of 0.91 indicates that the proposed MEMS gravimeter has successfully observed the Earth tides.

**Fig. 6:**
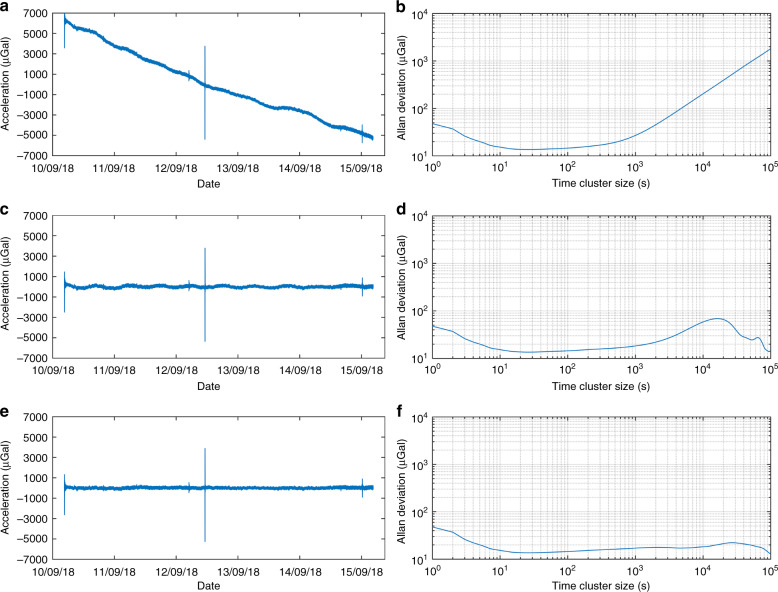
Allan deviation. **a** The raw data with a sampling rate of 1 Hz. **b** The Allan deviation of the data in a. **c** The raw data with temperature correction and removal of the linear drift. **d** The Allan deviation of the data in **c**. **e** The data in **c** with removal of the Earth tides. **f** The Allan deviation of the data in **e**

Allan deviation^41^ was used to analyse the data to investigate the stability characteristics. Figure 6a shows the raw data with a sampling rate of 1 Hz, while Fig. 6b shows the Allan deviation of the raw data. Figure 6c shows the same data after a linear temperature correction and removal of the linear drift. Figure 6d shows the Allan deviation of the data in Fig. 6c. Figure 6e shows the data after removing the tide effect. Figure 6f shows the Allan deviation of the data in Fig. 6e. The peak in Fig. 6a, c, e on 12 September was identified as a tele-seismic signal caused by the earthquake that occurred in Hanzhong, Shanxi, China. According to the Allan deviation results, a bias instability of 13.5 μGal can be obtained with an integration time of 20 s.

## Discussion

The performance parameters of a commercial CG-5 relative gravimeter, the previously reported MEMS gravimeter, and the MEMS gravimeter proposed in this paper are listed in Table 2. It is worth noting that the sensitivity has generally been used to represent the noise floor of a gravimeter in the geophysical field. Since the overall sensitivity of these two MEMS gravimeters is dominated by the displacement transducer, the proposed MEMS gravimeter with a sensitivity of 8 μGal/√Hz is five times more sensitive than the reported MEMS gravimeter. Moreover, benefitting from the larger working ranges of both the optical displacement transducer and the curved-beam-based suspension, the proposed MEMS gravimeter has a dynamic range of 8000 mGal, which is comparable with that of the CG-5 gravimeter and eight times larger than that of the reported MEMS gravimeter; therefore, this proposed MEMS gravimeter is promising for measuring gravity variations worldwide.

**Table 2:** Performance comparison between this work and other relative gravimeters

Parameters/devices	CG-5^16^	Ref. ^33^	This work
Fundamental frequency (Hz)	3	2.3	3.1
Sensitivity (μGal/√Hz@1 Hz)	2	40	8
Dynamic range (mGal)	8000	1000	8000
Displacement transducer (nm/√Hz@1 Hz)	0.07	2	0.2
Vacuum pressure (Pa)	/	10^−5^	1–10
Technology	Fused Silica	MEMS	MEMS

Moreover, to the best of our knowledge, this study is the first to perform co-site tide measurements using a MEMS gravimeter and a GWR iGrav superconducting gravimeter, which is the most sensitive commercial relative gravimeter. The strong correlation coefficient of 0.91 between our experimental data and the data acquired by the superconducting gravimeter indicates that the proposed MEMS gravimeter is qualified for use in earth tide measurements.

Based on the results of the Allan deviation, a gravity anomaly, due to an ore body with a residual density of 1.6 g/cm^3^ and a size of 40 × 40 × 40 m^3^ at a depth of 220 m, can be observed by the proposed MEMS gravimeter with an integration time of 20 s. In addition, based on the prior works in our laboratory, such as that on absolute gravimeters^14^, we can propose more applications. For large-scale deployment in the field, dozens of low-cost MEMS gravimeters, which should be regularly calibrated by an atom interferometry absolute gravimeter, can be used to prospect or to take time-dependent images of the local gravitational field for natural hazard forecasting.

In conclusion, we present a newly developed MEMS device that can measure earth tides, which has been qualified by using a GWR iGrav superconducting gravimeter for co-site tide measurements. The proposed MEMS gravimeter has a sensitivity of 8 μGal/√Hz and a dynamic range of 8000 mGal, which is on the same order as that of the commonly used commercial relative gravimeter CG-5; therefore, the proposed gravimeter is promising as a practical gravimeter that can be deployed worldwide for underground resource exploration, geophysics studies, geological hazard detection, and other applications.

## Materials and methods

### FEA simulation

The commercial FEA software COMSOL Multiphysics was used to simulate the nonlinear behaviour of the suspension. In the simulations, the Young’s modulus, Poisson’s ratio, and density of silicon were set to 129 GPa, 0.28, and 2330 kg/m^3^, respectively. Tetrahedral elements were used to mesh the structure. Boundary conditions were set with the suspension anchors fixed and a pre-defined displacement applied on the proof mass. The nonlinear solver was used to compute the model with the geometric nonlinearity option selected. When the pre-defined displacement sweeps over a specific range, the reaction forces applied on the anchors can be solved.

### Microfabrication

**Fig. 7:**
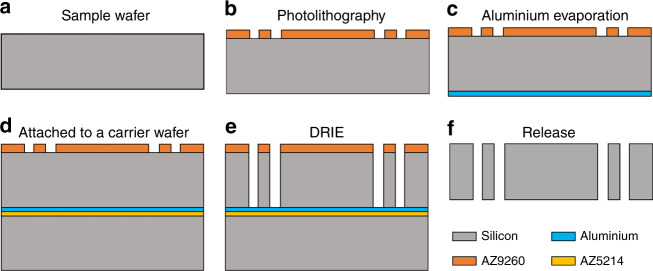
Fabrication process of the MEMS mechanism

A customized through-wafer deep reactive ion etching (DRIE) process was used to fabricate the MEMS mechanism, including the suspension, the proof mass, and the central slit simultaneously. The microfabrication process flow is shown in Fig. 7. The MEMS mechanism was based on a 4-inch 400-μm-thick single-crystal silicon wafer with a crystal orientation of <100>, and halo masking technology^42^ was used to design the photomask. First, the wafer surface was properly cleaned, followed by spin-coating of a 15-μm-thick photoresist layer (AZ9260). The photoresist was then patterned by a standard photolithography process. A 300-nm-thick aluminium layer was then evaporated onto the other side of the wafer. Then, the sample wafer was attached to a carrier wafer with the patterns facing up using spin-coated 2-µm-thick photoresist (AZ5214) as the adhesive layer. The sample was then placed on a hotplate at 100 °C for 2 min to ensure good adhesion between these two wafers. Afterwards, the sample was loaded into an Oxford Instruments PlasmaPro Estrelas100 to run a standard Bosch process for at least 60 min until the sample wafer was etched through. In the last step, the etched sample was soaked in acetone for at least 12 h to strip the remaining photoresist and de-attach the sample wafer from the carrier, followed by alkaline treatment to remove the aluminium layer. Benefiting from the dicing-free mask design, the MEMS device could be easily released from the wafer.

Several technologies were applied to achieve high fabrication quality. The application of halo masking technology reduced the micro-loading and etch-lag effects^43^, resulting in the same width of etching trenches. In addition, the backside deposited aluminium layer alleviated the notching effect of the through-wafer DRIE process. In this case, the trench profiles were nearly vertical, with a tolerance of ~1°. Moreover, the carrier wafer provided good mechanical support and heat conduction for the sample wafer during the DRIE process.

### Gravimeter assembly

The MEMS mechanism, the LED and the QPD were mounted on a U-shaped frame made of ultra-low thermal expansion (ULE) glass. First, the LED (Thorlabs LED630L) was glued on the glass frame. Then, the QPD (HAMAMATSU S5980) was aligned to the centre of the light spot by minimizing the difference in the photocurrent and then glued on the glass frame, followed by alignment and mounting of the MEMS mechanism with the same criteria. A thermistor was glued on the ULE frame near the MEMS mechanism to monitor the temperature changes. To avoid ambient temperature and pressure disturbances, the device was sealed in a vacuum chamber that was pumped to 1–10 Pa without the requirement of a getter or maintenance. Then, the chamber was shielded by thermal insulation materials to further alleviate the effects due to temperature changes and airflow. The LED was driven by a waveform generator (Keysight 33600 A) with a driving frequency of 666 Hz and a duty circle of 50%. The photocurrents were converted by a customized TIA circuit with a gain of 10^7^ V/A. The pre-amplified analogue signal was demodulated by an analogue demodulator on the same PCB board. Then, the demodulated signal was passed through a low-pass filter with a cut-off frequency of 5 Hz. Finally, the amplified signal was recorded by a 24-bit analogue-to-digital convertor (National Instruments PXI-4462).

### Tide signal processing

The outputs of the MEMS gravimeter and the thermistor were recorded for a long time with a sampling rate of 1 Hz. The raw data of the gravimeter are shown in Fig. 6a. The regression method was used to identify the linear drift (2.4 mGal/day) and the temperature coefficient (55 μGal/mK). Then, the linear drift and temperature variation effects were removed from the raw data, as shown in Fig. 6c. The corrected data were processed by a low-pass filter with a cutoff frequency of 0.062 Hz, which was also applied to the data of the superconducting gravimeter. Then, the data were averaged with a time constant of 60 min, as shown in Fig. 5. The correlation coefficient between the data in Fig. 5 was obtained using the MATLAB ‘*corrcoeff*’ function.

## Supplementary information

Supplementary Information

## Data Availability

The data used in this manuscript are available from the corresponding author upon reasonable request.
