# Supplementary material for: A high-sensitivity MEMS gravimeter with a large dynamic range
Source: Microsyst Nanoeng. 2019 Oct 7;5:45. doi: 10.1038/s41378-019-0089-7 (PMC6799805; doi:10.1038/s41378-019-0089-7)
Supplement: Supplementary file 1 — Supplementary Information [file 41378_2019_89_MOESM1_ESM.docx]

# *Microsystems & Nanoengineering*

Sensors: A MEMS gravimeter combines high precision with portability

Researchers in China have developed a small, portable gravimeter based on micro-electromechanical-system (MEMS) technology with a sensitivity and dynamic range comparable with larger, commercially available gravimeters. The new device was created by a team led by Liangcheng Tu at Huazhong University of Science and Technology. The MEMS mechanism consists of a spring-mass system designed around a combination of curved and folded beams. Together, these ensure that the system is stiff under low loads but flexible at loads around 1 g. An optical component measures the displacement of the proof mass to measure gravitational acceleration. The team tested their design alongside a commercial gravimeter and found a 90% correlation in the measurements of Earth tides, demonstrating its utility for applications from oil and gas exploration to hazard detection.

Related article manuscript number: MICRONANO-00757R1

Article title: A HIGH-SENSITIVITY MEMS GRAVIMETER WITH LARGE DYNAMIC RANGE

Corresponding author and affiliation/s: Liangcheng TU, Huazhong University of Science and Technology, School of Physics, Wuhan, Hubei, China

**About your Editorial Summary — please read**

**Before approving this Editorial Summary, please carefully check that (1) the summary text lists the correct author(s) and (2) the spelling and order of all author names and affiliations are correct.**

This **Editorial Summary** is based on your manuscript that was recently accepted for publication in *Microsystems & Nanoengineering*. It provides a non-specialist audience with a synopsis of your key research outcomes and conclusions. This value-added service provided by Springer Nature is designed to raise interest in your research across the broader community.

Springer Nature will publish the summary on the journal’s website, and it will be freely available under a under the CC BY licence (Creative Commons Attribution v4.0 International Licence) (see the journal website for details). We encourage you to re-use the summary to bring attention to your research; for example, you can host it on your own website and share it via social-networking platforms. Please attribute the summary to *Microsystems & Nanoengineering* and your article (e.g. by providing a link to your article) and do not make derivatives.

Please note that to maximise the usefulness of these summaries they must follow several stringent guidelines:
-- Spelling, punctuation and style are set according to *Nature* editorial guidelines. As this summary is aimed at non-expert readers, some concepts and technical terms will be simplified.
-- Total length must be no more than 135 words. It is likely that not all points in the paper will be covered.
-- The first sentence must be no more than 280 characters, including spaces, to allow use on microblogging sites.
-- The headline must consist of a brief generic subject identifier followed by a short description. No more than 10 words in total.

Please contact the editorial office ([mems_nano@mail.ie.ac.cn](mailto:mems_nano@mail.ie.ac.cn)) immediately with corrections should you find any factual errors in this Editorial Summary.
